# Predictive Factors for Lower Limb Amputation in Type 2 Diabetics

**DOI:** 10.7759/cureus.39987

**Published:** 2023-06-05

**Authors:** Sawsen Nouira, Taïeb Ach, Foued Bellazreg, Asma Ben Abdelkrim

**Affiliations:** 1 Department of Endocrinology, University Hospital of Farhat Hached, Sousse, TUN; 2 Department of Infectious Diseases, University Hospital of Farhat Hached, Sousse, TUN

**Keywords:** diabetic foot ulcer management, lower limb amputation, peripheral arterial diseases, peripheral sensory neuropathy, type 2 diabetes mellitus

## Abstract

Introduction: Type 2 diabetes mellitus (T2DM) is a major public health problem. Foot-related complications are common in diabetic patients. The aim of this study is to identify predictive factors for lower limb amputation (LLA) in order to better identify this at-risk population.

Methods: This was a cross-sectional study involving 134 patients who were hospitalised for the management of T2DM complicated by diabetic foot, in the department of endocrinology and diabetology. We included patients with T2DM whose diabetes was diagnosed 10 years ago or more, and who had a diabetic foot problem. Statistical differences between predictors of amputations were tested using: t-tests for numerical variables and chi-square tests for categorical variables. Significant variables were analysed by logistic regression to determine significant predictors.

Results: The mean duration of diabetes was 17±7 years. We found that 70% of patients with LLA were older than 50 years (p<10-3). The prevalence of LLA was higher (p=0.015) in patients with diabetes for more than 20 years. We noted that 58% of patients who underwent LLA were hypertensive (p<10-3). The majority of patients with LLA (58%) had abnormal micro-albuminuria (p<10-3). We found that 70% (n=12) of patients with LLA had low-density protein cholesterol levels above the target value (p<10^-3^). Diabetic foot grade ≥4 (4 or 5) according to Wagner's classification, was present in 24% of amputee patients. Based on a 95% confidence interval level, the independent significant predictive factors for LLA in our patients were: T2DM for more than 20 years, hypertension and diabetic foot grade ≥4.

Conclusions: After multivariate analysis, the significant independent predictive factors associated with LLA were: T2DM for more than 20 years, hypertension, and diabetic foot grade ≥4. Early management of diabetic foot problems is therefore recommended to avoid amputations.

## Introduction

Type 2 diabetes mellitus (T2DM) is a major public health problem that has grown over the past two decades [1]. According to the International Diabetes Federation, in 2021, there were 537 million patients with diabetes, including 24 million in Africa, and this number is expected to rise to 643 million in 2030 and 783 million in 2045 [1]. Complications associated with T2DM include macrovascular conditions such as coronary artery disease and peripheral artery disease (PAD), and microvascular complications, such as nephropathy, retinopathy and peripheral neuropathy [2]. Diabetic foot problems (DFP) are very common. This risk is due to the coexistence of peripheral neuropathy, infection and the presence of PAD in the lower limbs, which is thought to cause an alteration in the scarring process [3]. The main complications are diabetic foot ulcerations (DFU), skeletal deformities of the foot (Charcot foot) and amputations [4]. According to the International Working Group on the Diabetic Foot (IWGDF), DFU are wounds penetrating through the dermis and located below the ankle in a diabetic patient [5]. DFU cause significant suffering for the patient and family, and are also a considerable burden to society and health care institutions [6].

DFU are predictive of high mortality according to the International Diabetic Foot Task Force, with an estimated 5% mortality in the first 12 months of diagnosis and 42% mortality after 5 years of progression [7]. DFP can result in minor or major amputation, with a significant impact on patient's life expectancy and quality of life. Non-traumatic limb amputations are attributed in 70% of cases to diabetes [7]. However, according to the World Health Organization (WHO), half (50%) of amputations are avoidable with appropriate screening and management.

Given the challenges associated with amputation in diabetic patients, the identification of its predictive factors would be crucial. Diabetologists and primary care physicians need to identify the predictive factors for lower limb amputation (LLA), in order to provide appropriate prevention of these injuries.

The aim of this study is to identify the predictive factors of LLA in patients who presented with a DFP.

## Materials and methods

A cross-sectional study was carried out involving 134 patients who were hospitalised for management of T2DM complicated by DFP, in the department of endocrinology and diabetology. We used a questionnaire containing information on the socio-demographic and clinical characteristics of patients with T2DM. Before administering the questionnaire, participants gave written informed consent.

We included patients with T2DM whose diabetes was diagnosed 10 years ago or more who had a DFP. A duration of 10 years or more was chosen because this is the average time after which the degenerative complications of diabetes set in.

Several clinical aspects were evaluated to determine the relationship with LLA. Different classification systems have been employed to evaluate the severity of DFP. These systems are based on different characteristics of the ulcer, including depth, size, neuropathy, ischemia, and infection. The Wagner-Meggit system classification is one of the most widely used ones (Table 1) [8].

**Table 1:** Wagner's diabetic foot classification

Foot grade	Characteristics
0	high-risk foot
1	superficial ulcer
2	deep ulcer, penetrating tendon, bone or joint
3	deep ulcer with abscess or osteomyelitis
4	localised toe, forefoot or heel gangrene
5	hindfoot gangrene

Other factors were analysed: age, gender, duration of T2DM, smoking habits, hypertension, hyperlipidaemia, body mass index (BMI), renal profile, therapeutic strategies, PAD and neuropathy.

According to the classification adopted by the WHO [9], obesity is defined as a BMI greater than 30 kg/m².

Peripheral vascular evaluation was performed using a Doppler ultrasound machine. An ankle-arm systolic value of less than 0.8 was used to indicate the presence of PAD.

Sensory neuropathy was assessed using the 10 g monofilament (Semmes-Weinstein). The four-question neuropathic pain questionnaire (DN4) was evaluated [10]. The diagnosis of peripheral sensory neuropathy was made in the presence of a DN4 ≥4/10 and/or a pathological monofilament test and/or suppression of the tendon reflexes [10].

Statistical differences between predictors of amputations were tested using: t-tests for numerical variables and chi-square tests for categorical variables. A p-value <0.05 indicates statistical significance.

Significant variables were analysed by logistic regression to determine significant predictors. Statistical analysis was performed using SPSS version 21.0.

## Results

Our study included 134 patients, 14% of whom had a history of LLA prior to hospitalisation and 12% (n=17) had LLA during the study period. The mean age of our patients was 59±11 years, with extremes ranging from 25 to 85 years, and male predominance (n=68). A large number of our patients (14%) had a history of LLA before hospitalisation. We found that 70% of patients with LLA were older than 50 years (Table 2).

**Table 2:** Characteristics of the patients with type 2 diabetes mellitus, according to whether they underwent or not a lower limb amputation. *p-value calculated using Fisher’s exact test

Characteristics		Non-amputation n=117	Amputation n=17	*p‑value
Age (year)	<50 (n=22)	17	5	<10^-3^
	≥50 (n=112)	100	12	
Obesity	Yes (n=87)	77	10	<10^-3^
	No (n=47)	40	7	
Hypertension	Yes (n=70)	60	10	<10^-3^
	No (n=64)	57	7	
Smoker	Yes (n=48)	38	10	0.034
	No (n=86)	79	7	

The mean duration of diabetes was 17±7 years, with extremes (10 to 50 years). This duration was greater than 20 years in 51 cases. We found that the number of patients with amputations was significantly (p=0.015) higher in patients with diabetes for more than 20 years.

We categorised DFP according to Wagner's classification (n=134), 16 (12%) were grade 1, 11 (8%) were grade 2, 75 (56%) were grade 3, 15 (11%) were grade 4 and 17 (13%) were grade 5.

Obesity was more prevalent in patients with LLA (58% (n=10), p<10-3). We noted that 58% of patients who underwent LLA were hypertensive. The mean blood pressure was significantly higher in patients with LLA (p=0.003). Smoking was significantly associated with LLA (p=0.03) (Table 2).

More than half of patients with LLA (58%) had abnormal micro-albuminuria (p<10-3). We found that 70% (n=12) of patients with LLA had low-density lipoprotein (LDL) cholesterol levels above the target value (p<10-3). Clinical signs of diabetic neuropathy were present in all patients with LLA. Nephropathy and retinopathy were present in most (70%, n=12) of the patients who underwent LLA.

There was a lower risk of LLA in patients who were treated with insulin (47% vs 52%, <10-3), as well as in patients who were on statin (35% vs 64%, p<10-3) (Table 3).

**Table 3:** Characteristics of type 2 diabetes mellitus (T2DM) in the patients (n=134), according to whether they underwent or not a lower limb amputation. *p-value calculated using Fisher’s exact test LDL-C: low-density lipoprotein cholesterol

Characteristics		Non-amputation n=117	Amputation n=17	*p‑value
Duration of T2DM (year)	<20 (n=83)	77	6	0.015
	≥20 (n=51)	40	11	
Medication	Insulin (n=72)	64	8	<10^-3^
	Statin (n=42)	36	6	<10^-3^
LDL-C	Above target (n=84)	72	12	<10^-3^
	Within target (n=50)	45	5	
Diabetic nephropathy	Yes (n=84)	72	12	<10^-3^
	No (n=50)	45	5	
Diabetic retinopathy	Yes (n=102)	90	12	<10^-3^
	No (n=32)	27	5	
Peripheral artery disease	Yes (n=34)	24	10	10^-3^
	No (n=100)	93	7	
Cerebrovascular accident	Yes (n=20)	14	6	0.012
	No (n=114)	103	11	
Coronary heart disease	Yes (n=20)	16	4	<10^-3^
	No (n=114)	101	13	

To determine the significant predictors for LLA, we considered the following factors to be clinically important: age of the patient ≥50 years, obesity, hypertension, duration of T2DM over 20 years, insulin therapy, statin intake, LDL level, nephropathy, retinopathy, PAD and Wagner's classification (grade ≤3 vs grade ≥4). Based on a 95% confidence interval level, the independent significant predictive factors for LLA in our patients were: T2DM for more than 20 years, hypertension and a diabetic foot grade ≥4 according to Wagner's classification (Table 4).

**Table 4:** Results of the stepwise logistic regression analysis *p-value calculated using Fisher’s exact test **according to Wagner's classification OR: odds ratio; CI: confidence interval; T2DM: Type 2 diabetes mellitus

	OR	95% CI	*p‑value
Duration of T2DM (year) ≥20	3	0.00-11	<10^-3^
Hypertension	4	1.08-29	0.036
diabetic foot grade ≥4**	9	2-48	0.003

## Discussion

DFU are the most common and severe complications of diabetes and are the main cause of hospitalisation worldwide [11]. Previous studies have shown that T2DM increases the risk of LLA by 20-fold, resulting in disability, impaired quality of life and increased medical expenses [12]. In our study, we found that 14% of our patients had a history of LLA prior to hospitalisation and 12% had LLA during the study period. This result is lower than the rates reported by other studies. Nather et al., reported an amputation rate of 27.2% [13], while Leung et al., reported an amputation rate of 30.3% [14]. An earlier study by Harwant et al., in Kuala Lumpur Hospital, Malaysia, found an amputation rate of 20% [15].

Several risk factors have been widely studied and have been associated with diabetes-related LLA. We found that 70% of the patients, who had LLA, were older than 50 years, (p<10-3). Our results are consistent with those of Gandhi et al., who noted that the incidence rate of all diabetes-related LLA increases with age in both sexes [16].

We found that the number of patients with amputations was significantly higher in patients with diabetes for more than 20 years (p=0.015). Our results are not in agreement with those of Mizouri et al., who found no correlation between the duration of diabetes and the grade of the diabetic foot (p=0.24) [17]. A multicentre study including 6487 diabetic patients showed an association between the duration of diabetes and the presence of neuropathy [18]. A systematic review reported the presence of a positive association between DFP and the duration of diabetes in eight studies [19].

The predictive factor for LLA may not be the patient's age itself but the duration of diabetes, a factor that is of course correlated with the patient's age in many cases. This hypothesis is supported by the results of studies in which the association between duration of diabetes and DFP was assessed and showed a positive association, even after age adjustment. Monteiro-Soares et al. reported this association between the development of DFP and the duration of diabetes [20].

According to our results, hypertension is a predictive factor for LLA in patients followed for T2DM (OR=5; p=0.004). Our results are not consistent with those of Gürlek et al. and Chou et al. [21, 22]. More than half (58%) of the patients with amputation were obese (p<10-3). Our results are in accordance with those of a cross-sectional study including 218 patients with T2DM who were admitted to Tengku Ampuan Hospital in Malaysia for diabetic foot problems, obesity was not a predictive factor for LLA [22].

Smoking was significantly associated with LLA in our study (p=0.03). According to the results of the multivariate analysis, it is not an independent predictive factor for LLA. A prospective study involving 776 patients showed that smoking did not increase the rate of amputation (p-value=0.61) [23].

Nephropathy and retinopathy were present in most (70%) of the patients with LLA. According to our results, the presence of these two complications is not a predictive factor for amputation. In the cross-sectional study by Yusof et al., retinopathy was not predictive of LLA, but nephropathy and elevated urea and creatinine levels were significantly associated with LLA [24]. Ndip et al. described the association between diabetic foot disease in people with advanced kidney disease and those on renal dialysis [25]. Predictive factors for DFU in patients with diabetic nephropathy include susceptibility to infection, as uraemia compromises many aspects of the defence mechanisms against infection [26]. The anaemia associated with chronic kidney disease is associated with poor tissue oxygenation and impaired wound healing.

In our study, we found that PAD was significantly associated with diabetic foot amputation. However, this complication was not a significant independent predictive factor for LLA. Our results are consistent with a study by Hussain et al., which showed that LLA rates did not change significantly in the overall study population and in the population with PAD [27].

According to our multivariate analysis, a diabetic foot grade ≥4 according to Wagner's classification is a predictive factor of LLA. A meta-analysis conducted by Chunmei et al., of about 21 studies involving 6505 participants, including 2006 patients who underwent LLA, concluded that gangrene classifying the diabetic foot by a grade ≥4 was a predictive factor for LLA (OR = 10.90, 95% CI = 5.73~20.8, P<0.00001) [28].

Despite the relevance of our results, our study has some limitations, notably the lack of evaluation of biological data that could be predictive factors of LLA, particularly glycated haemoglobin, markers of inflammation, and infection.

## Conclusions

T2DM is a disease that is increasing in frequency worldwide. It is therefore one of the major public health problems. DFU is very common and, in the most severe cases, can lead to LLA.

Our study showed that a duration of T2DM of ≥20 years, hypertension and diabetic foot grade ≥4 according to Wagner's classification were predictive factors for LLA in patients admitted with DFP. Thus, we recommend routine foot examination in patients with T2DM, and patient education on foot care measures which is essential to prevent the development of DFP, as well as for the early detection of these conditions.
